# Inhibition of Aurora Kinase B activity disrupts development and differentiation of salivary glands

**DOI:** 10.1038/s41420-020-00393-w

**Published:** 2021-01-18

**Authors:** Abeer K. Shaalan, Tathyane H. N. Teshima, Abigail S. Tucker, Gordon B. Proctor

**Affiliations:** 1 Centre for Host-Microbiome Interactions, Guy’s Hospital, Faculty of Dentistry, Oral & Craniofacial Sciences, King’s College London, London, UK; 2 Centre for Craniofacial and Regenerative Biology, Guy’s Hospital, Faculty of Dentistry, Oral and Craniofacial Sciences, King’s College London, London, UK; 3 Department of Oral Medicine, UCL Eastman Dental Institute, London, London, UK

**Keywords:** Cell proliferation, Senescence

## Abstract

Little is known about the key molecules that regulate cell division during organogenesis. Here we determine the role of the cell cycle promoter aurora kinase B (AURKB) during development, using embryonic salivary glands (E-SGs) as a model. AURKB is a serine/threonine kinase that regulates key events in mitosis, which makes it an attractive target for tailored anticancer therapy. Many reports have elaborated on the role of AURKB in neoplasia and cancer; however, no previous study has shown its role during organ development. Our previous experiments have highlighted the essential requirement for AURKB during adult exocrine regeneration. To investigate if AURKB is similarly required for progression during embryonic development, we pharmacologically inhibited AURKB in developing submandibular glands (SMGs) at embryonic day (E)13.5 and E16.5, using the highly potent and selective drug Barasertib. Inhibition of AURKB interfered with the expansion of the embryonic buds. Interestingly, this effect on SMG development was also seen when the mature explants (E16.5) were incubated for 24 h with another cell cycle inhibitor Aphidicolin. Barasertib prompted apoptosis, DNA damage and senescence, the markers of which (cleaved caspase 3, γH2AX, SA-βgal and p21, respectively), were predominantly seen in the developing buds. In addition to a reduction in cell cycling and proliferation of the epithelial cells in response to AURKB inhibition, Barasertib treatment led to an excessive generation of reactive oxygen species (ROS) that resulted in downregulation of the acinar differentiation marker Mist1. Importantly, inhibition of ROS was able to rescue this loss of identity, with Mist1 expression maintained despite loss of AURKB. Together, these data identify AURKB as a key molecule in supporting embryonic development and differentiation, while inhibiting senescence-inducing signals during organogenesis.

## Introduction

Aurora kinases are a family of serine/threonine kinases that are important regulators of cell division, functioning in the progression from mitotic entry to cytokinesis^1^. There are three Aurora kinases in mammals, Aurora kinase A (AURKA), Aurora kinase B and Aurora kinase C (AURKC). AURKA and AURKB catalyse critical phosphorylation events in mitosis while AURKC is mainly expressed in gametes and is important for meiosis^1^. AURKB, also known as AIM-1 or Stk-5, is a member of the chromosomal passenger complex (CPC), together with the inner centromere protein (INCENP), borealin and survivin^2^. AURKB is localised on centromeres from prophase to metaphase-anaphase transition, then re-localises to the spindle midzone and midbody from anaphase to cytokinesis^3^. AURKB drives cell division by promoting: (i) phosphorylation of histone H3 on Ser 10 (H3S10ph), an important histone posttranslational modification that regulates chromosome condensation (ii) chromosome bi‐orientation by correcting errors in kinetochore‐microtubule attachment, (iii) mitotic spindle checkpoint activation, (iv) control of sister chromatids, (v) cleavage furrow ingression, and (vi) cytokinesis^4^. In addition to histone substrates, AURKB can phosphorylate Hec1 protein to maintain the stabilisation of the central spindle^5^ and the cleavage furrow substrates: myosin II regulatory chain, vimentin, desmin and glial fibrillary acidic, to promote cytokinesis^6^.

AURKB is highly expressed in many tumours, such as non-small cell lung cancer, breast cancer, colorectal cancer, hepatocellular carcinoma, thyroid cancer and leukaemia^7^. Within the context of these cancers, it has been demonstrated that AURKB decreases the expression of p21^WAF/CIP1^, a cell cycle inhibitor, indirectly through suppressing p53 activity to facilitate cell cycle progression that antagonises apoptosis^8^. In addition, a recent study demonstrated that AURKB epigenetically upregulates the expression of *CCND1* which encodes cyclin D1, through phosphorylation of serine 10 on histone H3 (H3S10ph) at the promoter of *CCND1*^9^. Cyclin D1 forms active complexes with cyclin-dependent kinases 4/6 (CDK4/6) to phosphorylate retinoblastoma protein (pRb) to drive progression from G1 phase to S phase^10^. Although much progress has been made in elucidating the importance of AURKB in tumour biology, the involvement of AURKB in organ development remains largely unknown. This is surprising, given the well-acknowledged role of aurora kinases in modulating cell cycle progression and proliferation.

Branching morphogenesis is a conserved development mechanism by which organs such as lungs, pancreas, mammary and salivary glands increase their functional epithelial surface area to maximise secretion or absorption^11^. During branching morphogenesis, new branches form by budding or clefting. It has been shown that the formation of new buds during branching morphogenesis is associated with increased cell division, and treatments which reduce or abolish cell division, reduce or abolish branching morphogenesis^12^. These studies also revealed that differentiation of developing cells in the pancreas is highly dependent on cell division^12^. Despite its key contribution in regulating mitotic processes during cell division, the role of AURKB has not been investigated during organogenesis and development of branching organs.

The embryonic mouse SMG serves as an ideal system for studying the molecular mechanisms governing branching morphogenesis and differentiation. This is because the glands can be grown ex vivo on a floating filter at the air/media interface and can be manipulated genetically or pharmacologically while recapitulating the normal developmental processes that occur in vivo^13^. The earliest sign of murine SMG development is seen at E12 as a simple epithelial bud structure surrounded by condensed mesenchyme. This is followed by rapidly recurrent rounds of cleft formation and proliferation of newly formed epithelial buds, which characterise the early branching morphogenesis stages (E11–E14). During late development (E15–E18), secretory units begin to form that consist of differentiating secretory acinar cells^14^.

The current study is grounded on our previous work showing that AURKB is required for the functional and structural restoration of Mist1^+^ acinar cells following acute injury of adult murine glands^15^. To address the gap in understanding the physiological function of AURKB during development, we used ex vivo SMG organ culture and Barasertib, a highly potent and specific inhibitor of the enzymatic activity of AURKB^16^. We have identified that AURKB regulates cell division during early morphogenesis, in addition, it maintains proliferation and differentiation at later stages of development. Moreover, loss of AURKB during development upregulated DNA damage signals and ultimately directed the embryonic epithelial cells to apoptosis and senescence.

## Results

### AURKB regulates branching morphogenesis and bud expansion during early development

**Fig. 1:**
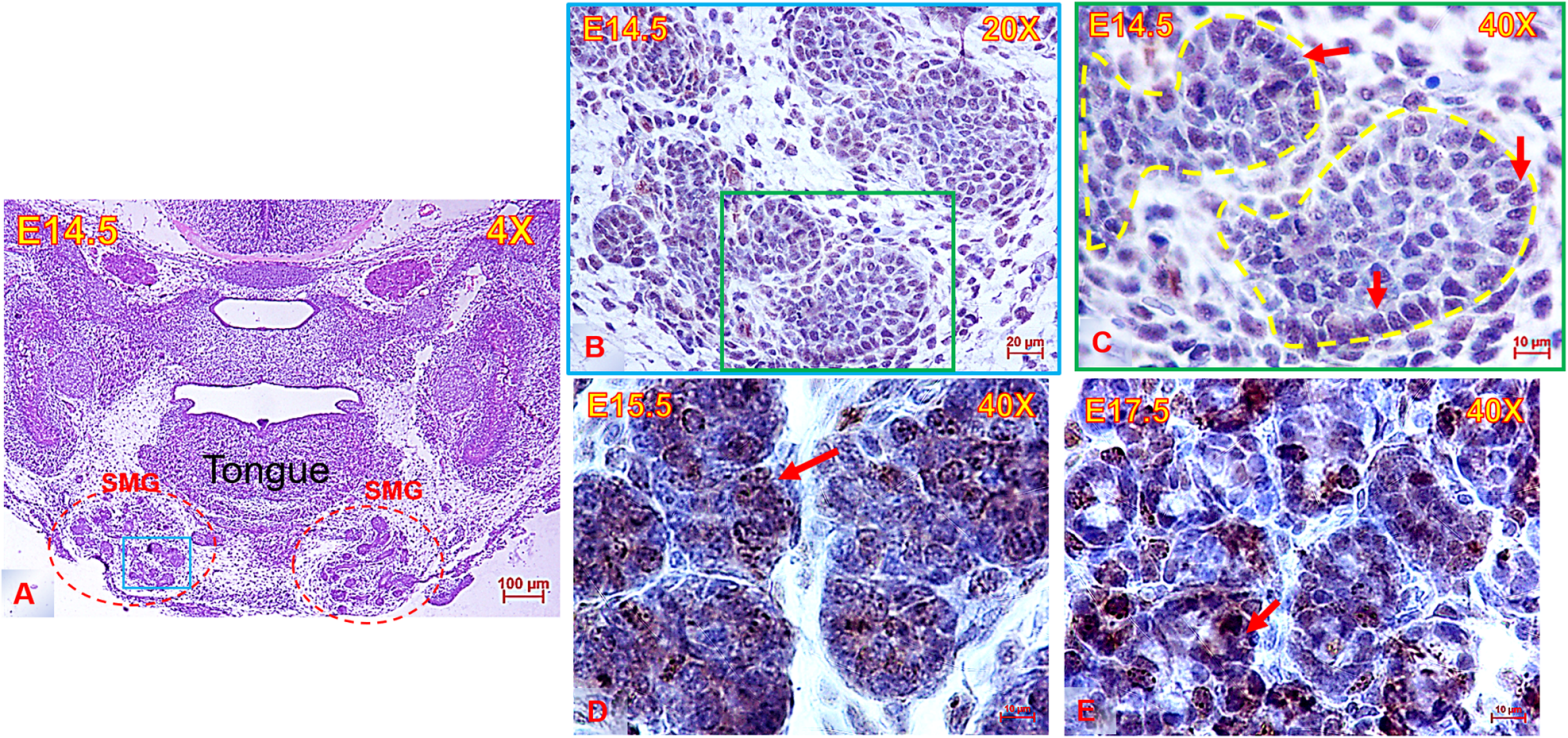
Anatomic location & histomorphology of the embryonic submandibular glands. **A** Low magnification image of E14.5 mouse head (frontal view) stained with H&E and showing the anatomical position of the submandibular glands (SMG) (red dotted outline) beneath the tongue, scale bar = 100 µm. Area outlined with blue square in (**A**) is stained with AURKB in (**B**), scale bar = 20 µm. **C**–**E** High magnification images showing abundant immunolabelling of nuclei with AURKB (brown) in the mouse SMGs throughout development. AURKB was mainly expressed in the bud nuclei, in addition to sporadic cytoplasmic immunostaining, scale bar = 10 µm.

In mitotic cells, AURKB is localised in the nucleus^17^. To elucidate if phospo-AURKB (active form) plays a potential role during E-SMG morphogenesis, we evaluated its localisation throughout different stages of development. The heads of mouse embryos were serially cut and stained with H&E to identify the sections with E-SMGs (example shown in Fig. 1A). Immunolabelling the embryonic glands with anti-AURKB (phospho T232) revealed consistent expression of the kinase during embryonic SMG development. As shown in Fig. 1B–F, AURKB (phospho T232) was mainly expressed in the nuclei of bud epithelial cells, in addition to sporadic staining of stromal cells. Similar staining patterns were detected in the adult murine SMGs^15^. To selectively inhibit AURKB in the developing SMGs, the highly specific, irreversible inhibitor Barasertib was used at a range of concentrations (120 nM–6 µM) in the incubation medium of ex vivo explant cultures at E13.5 and E16.5.

**Fig. 2:**
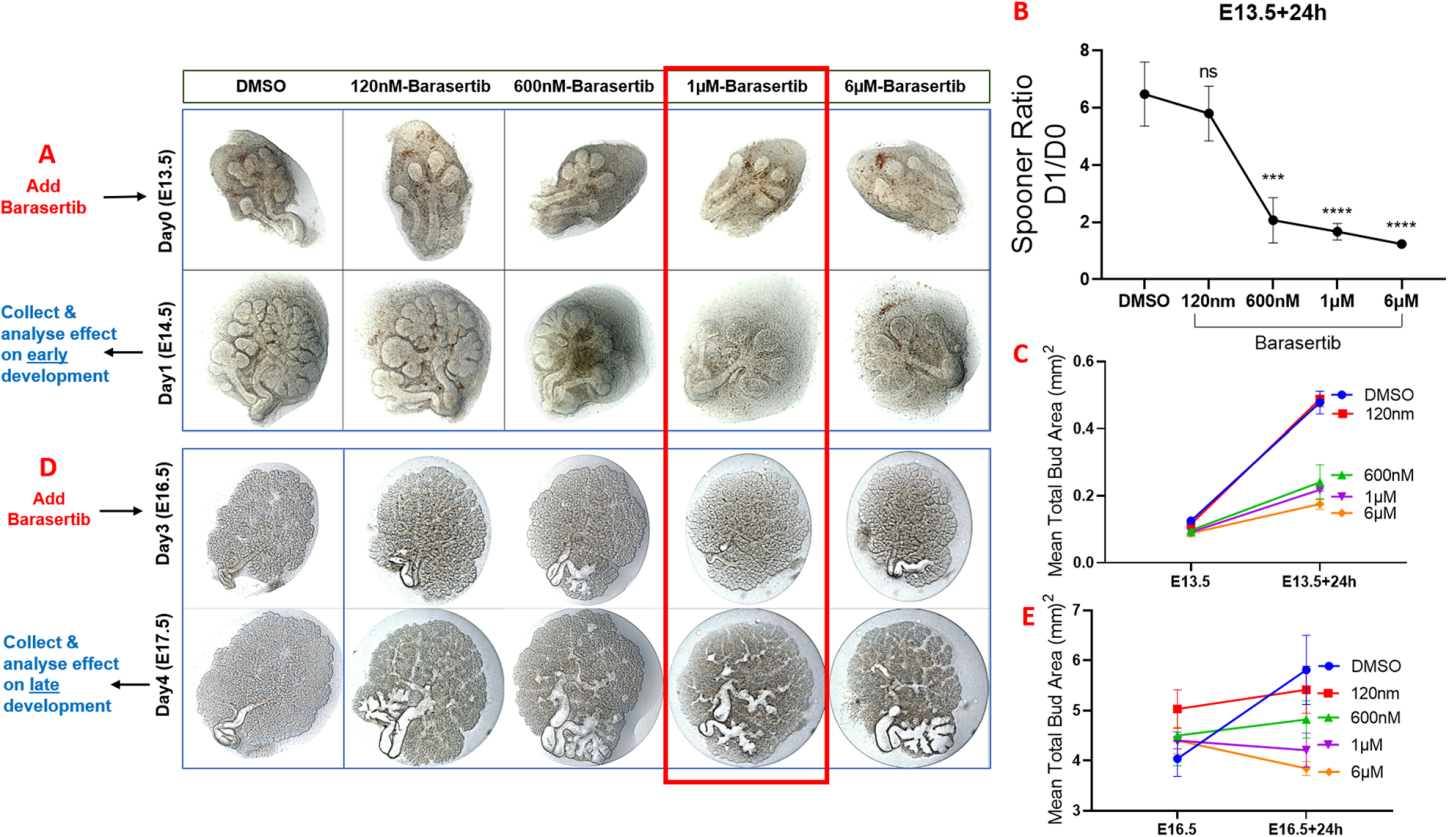
Inhibition of AURKB activity during early and late development of the embryonic submandibular glands. Inhibition of AURKB using Barasertib in the early (**A**–**C**) and late E-SMGs (**D**, **E**). Treatment of the explants with Barasertib for 24h reduced Spooner ratio in the early explants (**B**) and inhibited expansion of SMG buds in a dose-dependent manner (**C**, **E**). Analysis demonstrated gradual reduction of the mean total bud area with increasing doses of Barasertib, which was more clearly seen in the mature explants (late development) (**D**, **E**), *n* = 3 for each group. All downstream experiments were conducted using Barasertib at 1 µM (red outline).

**Fig. 3:**
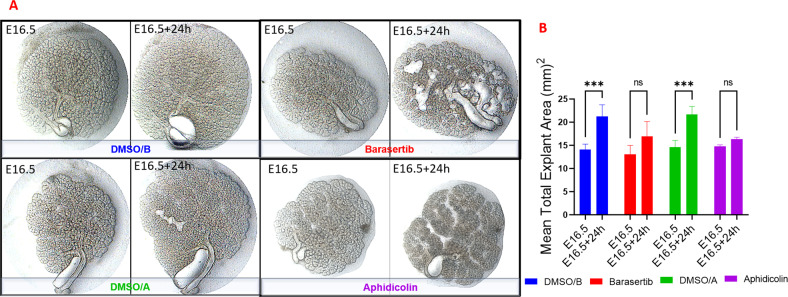
Impact of Barasertib (1 µM) on explant expansion compared to the cell cycle inhibitor Aphidicolin (500 ng/ml). Both inhibitors arrested growth of the SMG explants to the same extent (a, b), analysed by two-way ANOVA, ***P ≤ 0.001, ns: non-significant *p* > 0.05, *n* = 3 for each group.

These experiments revealed that inhibition of AURKB activity negatively impacted SG morphogenesis at both early (Fig. 2A–C) and late (Fig. 2D, E) development stages. Analysis of the Barasertib-inhibited explants revealed a dose-dependent reduction in the total bud area after 24 h compared to the DMSO-treated controls (Fig. 2C, E), with a more extreme inhibition of branching observed after 48 h (Supplementary Fig. 2). Based on these experiments, 1 µM Barasertib was chosen for downstream experiments since it resulted in arrested growth of the developing buds over 24 h. (Fig. 2E). To explore if the phenotypes presented are specific to AURKB-related cell cycle roles, the E16.5 glands were incubated with another cell cycle inhibitor Aphidicolin, which interferes with DNA replication and leads to DNA damage and apoptosis by inhibiting replicative DNA polymerases^18^. Aphidicolin diminished the growth of the explants to a level comparable to Barasertib (Fig. 3A, B).

### Histomorphologic alterations in the Barasertib-treated explants

**Fig. 4:**
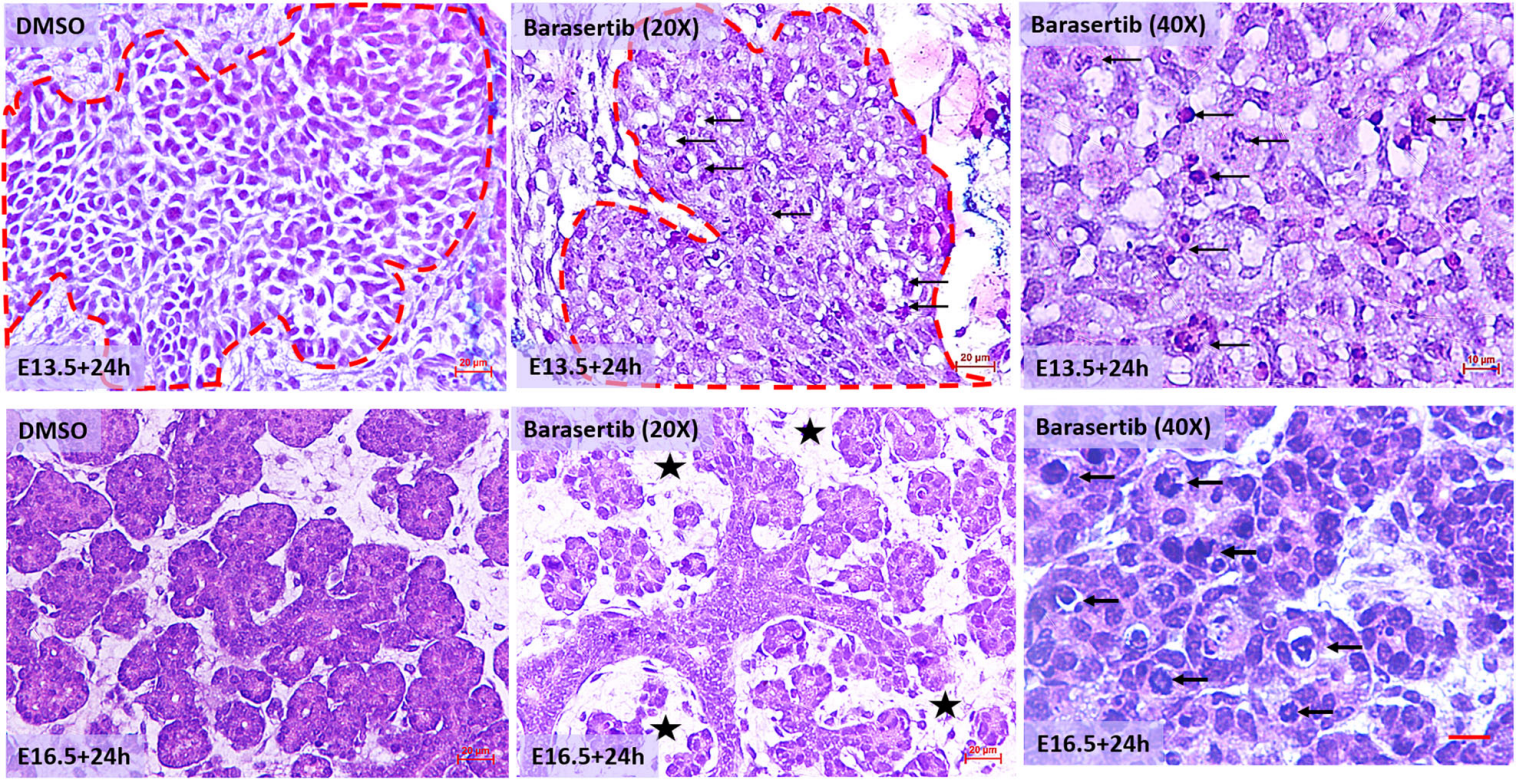
H&E staining of E13.5+24 h (epithelial buds are outlined in red dotted line) and E16.5+24 h glands treated with DMSO or Barasertib. Low magnification images show hypocellularity and increased mesenchymal spaces (asterisk) between the developing epithelia in the mature explants (E16.5 + 24 h), scale bar = 20 µm. High magnification images show abundant, enlarged, hyperchromatic nuclei in response to AURKB inhibition during early (E13.5 + 24 h) and late (E16.5 + 24 h) embryonic development (arrows), scale bar = 10 µm.

When the cultures were assessed at the cellular level, the control explants demonstrated clusters of uniform bud cells exhibiting regular nuclei, interspersed within loose mesenchyme. Conversely, the AURKB-inhibited explants revealed hypocellularity, irregular cell shape and size and frequent, enlarged hyperchromatic nuclei (Fig. 4). Notably, all E16.5 explants incubated for 24 h in Barasertib exhibited significant gaps between the epithelial branches so that the mesenchyme between the forming lobes was evident (Fig. 4)

### AURKB blockade reduced cycling and proliferation in the developing SMGs

**Fig. 5:**
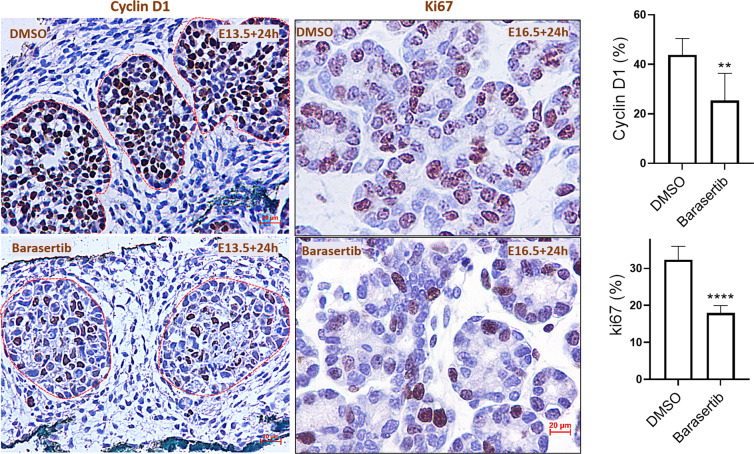
AURKB regulates bud cell cycling and proliferation during salivary gland development. Cyclin D1 and ki67 immunoexpression (brown staining) at E13.5+24h and E16.5+24h, respectively. Extensive, nuclear immunolabelling of the epithelial buds in the DMSO-treated buds, with sparse expression of both markers in the explants treated with Barasertib. Counting the percentage of immunopositive nuclei revealed nearly 50% loss in number of cycling (Cyclin D1) and proliferating cells (ki67), analysed by paired Student’s *t* test (**P ≤ 0.01, ****P ≤ 0.0001), scale bar = 20 µm.

Owing to the well acknowledged, canonical role of aurora kinases in driving the progression of the cell cycle and proliferation^7,9^, we investigated the level of Cyclin D1, a marker of cell cycle progression from the G1 to S phase^19^, after 24 h in culture. The ex vivo explants treated with the control vehicle displayed abundant Cyclin D1 nuclear staining in the peripheral and central epithelial bud cells, highlighting their active cycling during branching morphogenesis. Conversely, AURKB loss resulted in significant depletion in the percentage of Cyclin D1^+^ cells (Fig. 5). Notably, residual Cyclin D1 immunostaining was seen only in the central bud cells (Fig. 5). Likewise, Barasertib significantly diminished proliferation of the epithelial bud cells in the E16.5+24 h explants, as marked by a marked reduction of Ki67^+^ cells (Fig. 5).

### AURKB loss induced double-strand breaks (DSBs), apoptosis and senescence of the developing bud cells

**Fig. 6:**
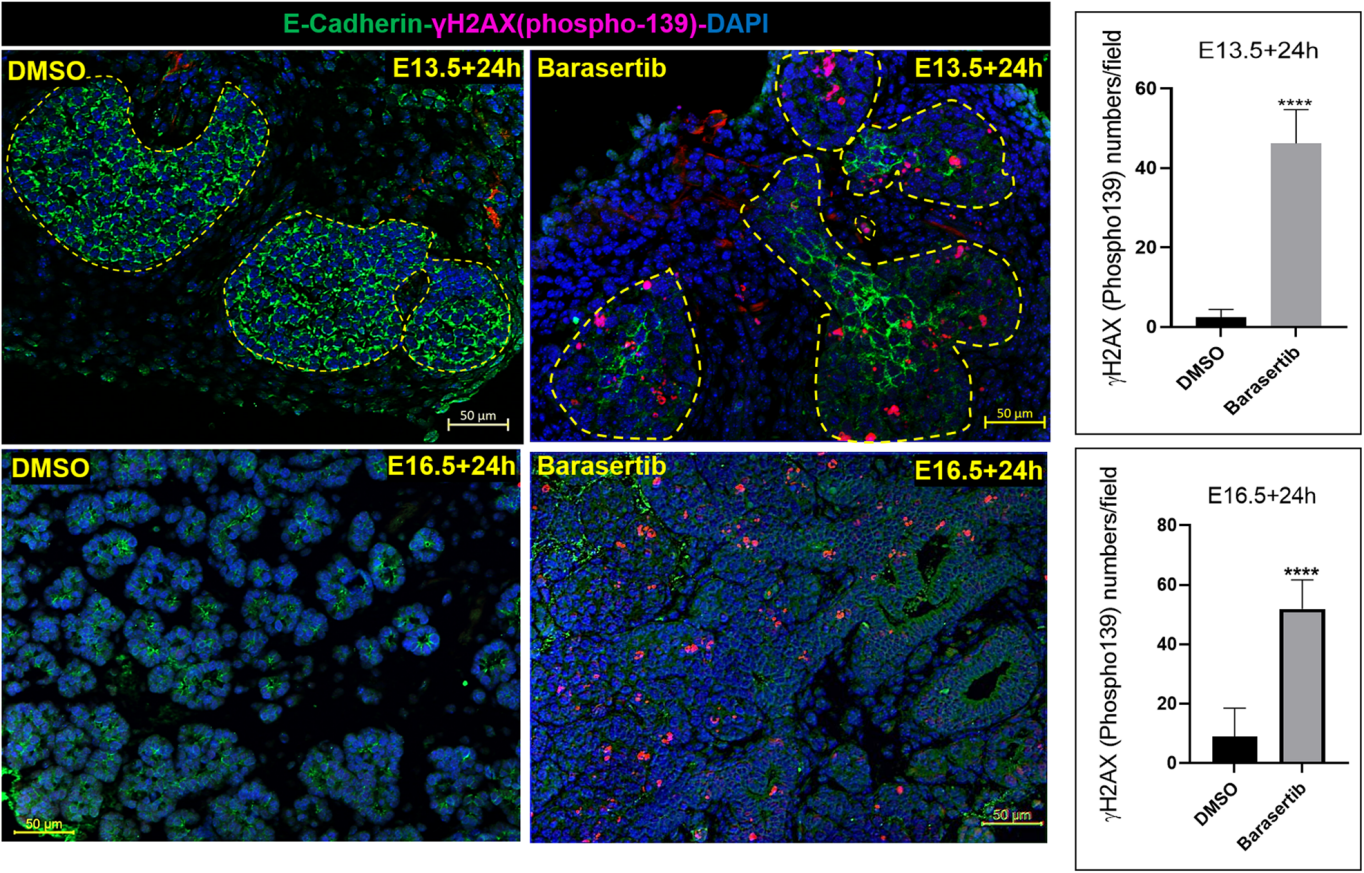
AURKB inhibition triggered double-strand breaks (DSBs): Abundant expression of phospho-γH2AX (magenta), a marker of double-strand DNA breaks, in the developing buds following AURKB blockade for 24 h. Cell counting revealed extremely significant increase in the number of bud cells exposed to double-strand breaks after AURKB inhibition, analysed by paired Student’s *t* test at E13.5 + 24 h and E16.5+24 h (****P ≤ 0.0001, number of glands analysed ≥ 3, number of fields/group =7 – 13), scale bar = 50 µm.

One of the consequences of knocking down AURKB is genomic instability^20,21^, which leads to severe or irreparable DNA damage, especially DNA DSBs^22^. The first protein to respond to DSBs is ataxia telangiectasia mutated (ATM), a kinase whose substrates include histone H2AX^23^. Phosphorylated H2AX (p.H2AX) rapidly localise to DSBs, forming characteristic foci in the nucleus^24^. The level of the phosphorylated form of γH2AX was therefore assessed in the cultures. The Barasertib-treated explants revealed numerous γH2AX^+^ foci in the epithelial buds after 24 h in culture, suggesting robust activation of DNA damage signalling (Fig. 6 and Supplementary Fig. 3). This indicated a rapid and robust DNA damage response (DDR) downstream of AURKB blockade during development.

**Fig. 7:**
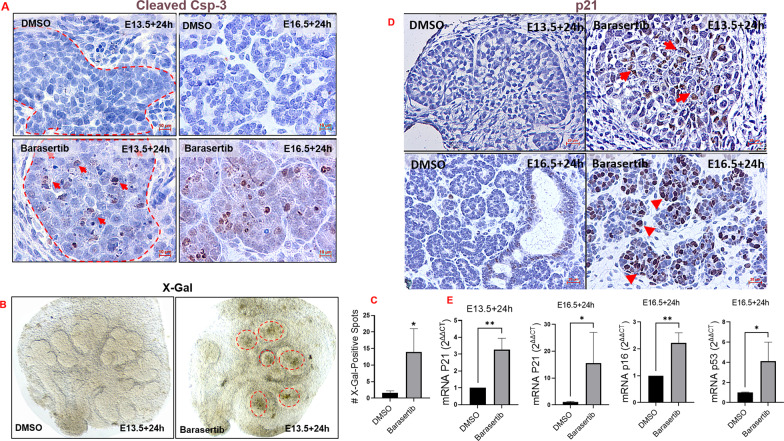
Inhibition of AURKB activity triggered apoptosis and senescence during early and late SMG development. **A** Cleaved caspase 3 (brown) immunolabelling bud cells undergoing apoptosis as a final fate following AURKB blockade for 24 h during early (E13.5+24h) and late (E16.5+24h) development, scale bar = 10 µm. **B**, **C** X-Gal assay performed on the SMG explants 24 h post DMSO or Barasertib treatments, shows significant accumulation of SA-βgal in the glands treated with the AURKB inhibitor (dotted outlines), analysed by Student’s *t* test (*P ≤ 0.05, *n* = 3 pairs). **D** Immunolabelling with p21 (brown staining; red arrows), 24 h post AURKB loss during early (E13.5+24h) and late (E16.5+24h) embryonic development. **E** Fold change of senescence-related genes: p21 was transcriptionally upregulated in both time points tested, p53 and p16 were only upregulated in the mature explants (E16.5+24h). Number of pooled glands ≥ 9 in E13.5+24h group and ≥3 in E16.5+24h obtained from three different experiments for each group. Gene expression was normalised to Gapdh and to corresponding experiment control groups and analysed by unpaired Student’s *t* test (*P ≤ 0.05, **P ≤ 0.01).

The DNA damage response triggered after aurora kinase inhibition may direct the cells to repair the break, undergo apoptosis, or become senescent^25^. Immunostaining for the apoptotic marker cleaved caspase-3 showed abundant epithelial expression following Barasertib treatment compared with controls (Fig. 7A). Apoptosis, therefore, was the final fate of some bud cells exposed to Barasertib. In addition, histologic analysis revealed the appearance of large cells with notably enlarged, hyperchromatic nuclei, morphologically reminiscent of cells undergoing senescence (Fig. 4).

To determine whether targeting AURKB in the highly proliferating, developing SG cells has triggered premature cellular senescence, we stained the embryonic wholemounts with X-Gal (5-bromo-4-chloro-3-indolyl-β-D-galactopyranoside) to detect senescence-associated β-galactosidase (SA-βgal), a well-documented marker of senescent cells^26^. Embryonic explants treated with Barasertib for 24 h showed substantially increased X-Gal activity which was located largely in the developing buds (Fig. 7B, C). In order to investigate the mechanism of Barasertib-induced senescence in the embryonic SMGs, we examined the expression levels of senescence-related genes/pathways in pooled explants treated with DMSO or Barasertib for 24 h at the two time points using RT-qPCR. E13.5 explants cultured for 24 h revealed that senescence during early embryonic development involved transcriptional upregulation of p21, which is activated by DNA damage responses^27^. The senescence-related gene profile during the cytodifferentiation stage varied in comparison to earlier development, featuring transcriptional upregulation of more senescence-related genes: p53 and p16 (Fig. 7E). To further substantiate that the senescence signal induced by AURKB loss was exclusively triggered in the developing bud epithelia, E14.5 and 17.5 glands incubated in DMSO or Barasertib were immunolabelled for p21, which was observed predominantly in bud cells (Fig. 7D), similar to X-Gal staining (Fig. 7B).

### Impact of AURKB loss on bud cell identity

**Fig. 8:**
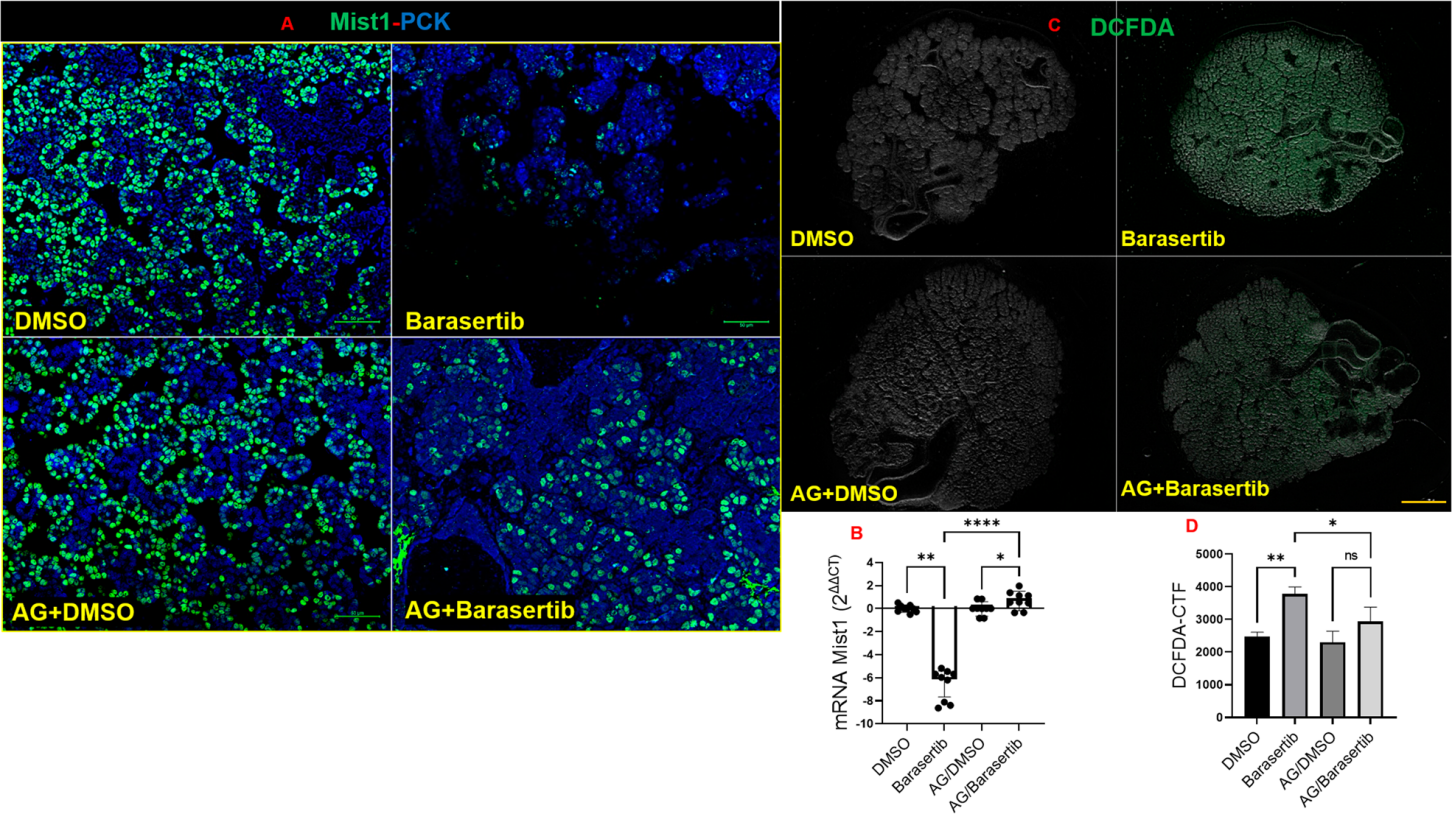
AURKB loss disrupted acinar identity via ROS generation. **A**, **B** Mist1 (green) and pan cytokeratin (blue) expressions in E16.5+24h bud cells, scale bar = 50 µm. Remarkable loss of the acinar differentiation marker Mist1 at the protein and transcriptional levels in response to Barasertib treatment. Recovery of Mist1 levels due to ROS scavenging using aminoguanidine (number of pooled glands ≥ 5), analysed by ANOVA **P ≤ 0.01, *p ≤ 0.05, ****P ≤ 0.0001. **C**, **D** ROS production in response to AURKB inhibition in the embryonic salivary glands. ROS levels were visualised in the SMG explants after staining with DCFDA and inspection under fluorescence microscope. Analysis of corrected total fluorescence (CTF) using ImageJ revealed that Barasertib treatment for 24 h significantly increased levels of ROS in the E16.5+24h explants which were significantly reduced after co-treating the explants with aminoguanidine, analysed by ANOVA, **P ≤ 0.01, *p ≤ 0.05, ns: non-significant, *p* > 0.05. Scale bar = 500 μm, *n* = 3/group.

Next, since the impact of Barasertib was mainly seen in the bud cells, we aimed to investigate the effect of AURKB blockade on levels of the acinar differentiation marker Mist1^28^ at the cytodifferentiation stage (equivalent to E16.5). At the transcriptional level, Mist1 did not change before and after incubation of the E16.5 explants in DMSO for 24 h (Supplementary Fig. 4A). Strikingly, Barasertib triggered significant loss of Mist1, at the transcriptional and protein levels (Fig. 8A, Supplementary Fig. 4B and Fig. 8B), suggesting that differentiation of the acinar cells had been altered. The link between AURKB and differentiation of the secretory epithelia has not previously been investigated, therefore we hypothesised that AURKB blockade may have initiated a damage signal that has altered the expression of the differentiation marker Mist1 in the acinar cells. To verify this hypothesis, we relied on cancer studies, which comprehensively characterise the array of damage signals downstream of AURKB inhibition. Results from these studies showed a functional relationship between AURKB inhibition and excessive generation of reactive oxygen species (ROS) in therapeutic modalities of cancer, as a mechanism of cytotoxicity induced by the aurora kinase inhibitor, Barasertib^29^. To visualise if ROS was produced in response to AURKB inhibition, we stained the DMSO/Barasertib-treated explants with DCFDA. These experiments revealed significant ROS generation in the Barasertib-treated explants compared to the controls (Fig. 8C, Supplementary Fig. 4C and Fig. 8D). To investigate the impact of ROS on the levels of Mist1 in the bud cells, aminoguanidine, a free-radical scavenger^30^ was included in the Barasertib-containing culture medium. Incubation of the SMG explants with aminoguanidine and Barasertib protected them against the generation of excessive ROS levels and maintained Mist1 at the transcriptional and protein levels (Fig. 8A–C).

## Discussion

There are few published studies that shed light on enhancers of cell division and proliferation during development. Herein, we demonstrate for the first time that AURKB is one of the key drivers of epithelial expansion during early and late organogenesis. Subcellularly, the active form of AURKB (Phospho-AURKB (T232)) was mainly seen in the nuclei of the developing bud cells and its inhibition resulted in a block of proliferation, DNA damage, apoptosis and senescence of these cells. To confirm the specific impact of AURKB on expansion of the bud compartment, we used Aphidicolin, a cell cycle inhibitor which arrested the overall growth of the explants in a manner similar to Barasertib. An important finding of the present study is the causal link demonstrated between AURKB loss and SG senescence. In our previous study^15^, adult SMGs were shown to constitutively express AURKB in various cell compartments. Senescent cells are present in aging salivary glands^31^ and compromise function following head and neck irradiation^32^. Results from this study suggest that dysregulated AURKB expression in the aged and irradiated glands may be an underlying mechanism driving SG senescence, which warrants further investigations.

Barasertib, has been extensively used by researchers as a specific inhibitor of AURKB. Barasertib inhibits cell growth in vivo in a dose-dependent manner in many cancers, including leukaemia, colorectal cancer, breast cancer, lung cancer, and some other solid tumours, and it is involved in phase II clinical trials^33^. In cultured cells, Barasertib-mediated inhibition of AURKB leads to failed biorientation of chromosomes and interferes with cytokinesis. As a consequence, polyploidy and eventual loss of viability occurs^34^. Similarly, the ex vivo embryonic SMG explants failed to grow in culture when incubated with high doses of Barasertib for 48 h (Supplementary Fig. 2). These results identify AURKB as an important regulator of embryonic cell division and imply a lack of redundancy with other kinases.

Cytoskeletal dynamics, epithelial motility as well as cell proliferation are critical for salivary gland branching morphogenesis^35^. During this intricate process, initial buds subdivide into multiple smaller buds that elongate and expand to form secondary buds. In our explant system, the control SMG explants contained many dividing cells in the developing buds, as evidenced by high expression of cell cycle and proliferation markers, Cyclin D1 and Ki67, respectively. Downregulation of these markers in response to AURKB inhibition paralleled the diminished ability of the embryonic glands to grow and expand. These results are in agreement with the inhibitory, anticancer effect of Barasertib on the expression of cyclin D1 and cell proliferation^9^.

The role of the cytoskeleton during branching morphogenesis has been identified in the developing mouse SMGs^36^. Despite its importance in targeting various cytoskeleton regulatory proteins including RhoA, Vimentin, Desmin and GFAP, the direct role of AURKB in salivary gland branching morphogenesis, has not been previously studied. Since incubation of SMG explants with Barsertib resulted in rapid failure of clefting and branching of the early E13.5 buds, we hypothesise that AURKB, in addition to its canonical functions in enhancing cell division, regulates salivary gland branching morphogenesis, via phosphorylation of several proteins that affect the structural organisation of intermediate filaments, microtubules and the actin cytoskeleton^37^. Future experiments will aim to highlight potential, non-cell cycle roles during E-SG development.

Following treatment with Barasertib, both the pseudoglandular and cytodifferentiated explants displayed enlarged and irregular-shaped nuclei, two features that are highly reminiscent of AURKB inhibitors in adult tissue^38^. Previous studies have shown that these irregular nuclei arise with AURKB inhibitors because cells enter M phase and condense their chromosomes, yet they eventually decondense without proper segregation and form mostly single and irregular-shaped nuclei^20^. These chromatin aberrations are reported to increase the DNA damage response^25^ and lead to cell death or cell cycle arrest in highly proliferating cells^39^. Our data demonstrate the formation of p.H2AX foci in the treated explants, suggesting that loss of AURKB rapidly predisposed the highly proliferating bud cells to double-strand DNA breaks (DSB).

Previous studies have shown that when genotoxic damage is irreversible, apoptosis and senescence become the designated fate to avoid propagation of genetically unstable progenies^40^. The dramatically stunted growth of the early buds (E13.5) incubated with Barasertib for 24 h, added to the abundant DSBs, suggests that cell cycle arrest and/or cell death signals were evoked in the developing embryonic cells. Indeed, immunolabelling of Barasertib-treated explants for the apoptotic marker; cleaved caspase 3, revealed that apoptotic cell death was triggered in many embryonic bud cells.

Cellular senescence refers to a process of permanent cell cycle arrest induced by various factors including telomerase deficiency, DNA damage, oxidative stress, oncogene mutation and many other intrinsic or extrinsic initiators^41^. These cause a DNA damage response (DDR), which leads to cell cycle block via stabilisation of p53 and transcriptional activation of p21, as well as the upregulated expression of p16, p19 and other signalling pathways^42^. Accumulation of senescence-associated β-galactosidase (SA-β-gal) and upregulation of senescence-associated genes p21, p16 and p53 in the E-SMGs verified the senescence phenotype triggered by Barasertib. Similar results were observed following AURKB inhibition of tumour growth^22^ and in irradiation-induced senescence of bone marrow mesenchymal stem cells^43^.

One of the remarkable outcomes of inhibiting AURKB in cytodifferentiated embryonic glands was the depletion of Mist1, a basic helix‐loop‐helix (bHLH) transcription factor that is expressed once cells have become committed to an acinar cell fate^44^. Importantly, Mist1 loss is also seen after the genotoxic stress triggered by salivary gland irradiation^45^. Therefore, Mist1 protein may be a downstream target of the DNA damage‐activated signalling in the salivary glands. Previous studies have demonstrated excessive ROS generation as one of the consequences of AURKB inhibition by Barasertib^29^. Our work demonstrates for the first time that AURKB functions in maintaining the redox status in embryonic cells, since its loss in the Barasertib-treated E-SMGs resulted in excessive ROS generation and loss of acinar cell identity by reducing the levels of key differentiation molecules.

Overexpression of aurora kinases has been shown to correlate with highly proliferative malignancy^46^ within the context of cancer. Although epithelial cell division is key during embryonic development, no reports are available on the roles played by AURKB in maintaining genomic stability of these highly proliferating tissues. By shedding light on the physiological contexts of aurora kinases, the present study along with some others^1,15,47,48^, raises awareness of the potential side effects of anticancer therapy based on the use of Aurora-specific inhibitors.

## Materials and methods

### Sample collection and ex vivo organ explant cultures

Mouse embryonic SGs were collected from CD1 animals housed in the Biological Services Unit at King’s College London. Mice were culled via schedule 1 culling methods, as approved by the Home Office and King’s College London. Animal experiments conform to ARRIVE (Animal Research: Reporting of In Vivo Experiments) guidelines. Glands were cultured in the presence and absence of AZD1152-HPQA (SML0268 Sigma) (referred to in the present article as Barasertib). Dissected submandibular and sublingual glands from mouse embryos were placed on permeable membranes (BD, Franklin Lakes, NJ, USA) over culture medium (DMEM—Advanced Dulbecco Modified Eagle Medium F12, Invitrogen, Waltham, MA, USA; 1% GlutaMAX, Invitrogen; 1% penicillin-streptomycin) at 37 °C and 5% CO_2_^49^. For functional experiments, embryonic murine explants at E13.5 or at E13.5+72 h were treated with Barasertib. For early inhibition, Barasertib (1 µM) was added to the freshly dissected E13.5 glands for 24 h in control medium. For exploring the effect of AURKB inhibition on late E-SMG development, the explants for the control and experimental groups were left in the control medium from E13.5 (date of excision) for three days (equivalent to E16.5), at which point Barasertib (1 µM) was added to the explant cultures for 24 h. For both time points, the negative control group, from the contralateral side of the embryo was incubated with the vehicle DMSO. Images of each gland were taken daily to follow development, and each group consisted of a minimum of three matched pairs of glands (*n* = 3–10 pairs per experiment). For branching analysis, the Spooner ratio (ratio between the number of buds found on the last day of culture divided by the number found at the start of culture) of treated groups was compared to contralateral control groups using a paired Student’s *t* test (GraphPad Prism Version 8 (GraphPad software, USA)). For bud area measurement, epithelial buds within each gland were outlined accurately using ImageJ, NIH^®^ ‘selection brush’ tool (Supplementary Fig. 1A and B). Scale was set for the images and the ‘area measurement’ command was used to measure the selected outlines. Spooner ratio for each tested group, before and after DMSO/Barasertib addition, were compared using Two Way ANOVA (GraphPad Prism Version 8 (GraphPad software, USA)). For cell cycle inhibition with Aphidicolin, E16.5 explants were incubated for 24 h with 500 ng/ml Aphidicolin (38966-21-1, Santa Cruz Biotechnology) for 24 h before harvest.

### RT-qPCR analysis

For RT-qPCR, the ex vivo explants were cultured in 1 µM of Barasertib for 24 h, then collected and stored in RNAlater^®^ (R0901-100ml, Sigma-Aldrich) till further use. To extract mRNA from the E14.5 and E17.5 tissues, the pooled explants (n = 10–13) were homogenised using a FastPrep™ tissue homogeniser (MP Biomedicals Santa Ana, CA) and RNeasy^®^ Micro Kit (74004, Qiagen) was used for total RNA extraction. RNA concentration as well as the A260/280 and A260/230 ratios were then measured with the NanoDrop ND-1000 Spectrophotometer (Thermo Fischer Scientific, Nottingham UK). iScript™ cDNA Synthesis kit (170–8890, Bio-Rad) was used to reverse transcribe 100 ng of extracted RNA. PCR reactions (10 µl/well) were prepared by adding SsoAdvanced™ Universal SYBR Green Supermix (172–5271, Bio-Rad), primers (PrimerDesign™, Ltd. mouse GAPDH and Qiagen QuantiTect Primer Assay, mouse Mist1, p21, p53 and p16) and cDNA template. Thermal cycling was performed using Corbett RotorGene 6000 System (Qiagen, UK). In all RT-qPCR experiments, fold change was assessed according to the following equation: 2^ΔΔCT^ = [Ct GOI Exp−Ct HKG Exp]−[Ct GOI Cal−Ct HKG Cal]: Ct: cycle threshold, GOI: gene of interest, Exp: poly (I:C)-injected glands, HKG: housekeeping gene, Cal: control glands injected by the vehicle. The values were analysed by GraphPad Prism Version 8 (GraphPad software, USA).

### Histology and Immunohistochemistry

Harvested explants were fixed in 4% paraformaldehyde solution for 20 mins then washed three times in phosphate buffered saline for 10 min each. Explants were processed and embedded in paraffin for long term storage. Histomorphological changes were examined using conventional H&E stain. For immunohistochemical studies, 3 µm tissue sections were deparaffinized, rehydrated, and unmasked in a single step using Trilogy™ (Cell Marque, Rocklin, CA, 920P-06). To block endogenous peroxidase activity and nonspecific background staining sections were incubated in 3% hydrogen peroxide solution for 20–30 min. To block all epitopes on the tissue samples and prevent nonspecific antibody binding, sections were incubated with 1% BSA in 1X TBS, pH 7.6 for 5 min. Tissue sections were incubated at 4 °C overnight, with the antibody: rabbit-anti-cleaved caspase 3 (1:2500, NB100–56113, Novus Bio), rabbit–anti-Mist1 (1:200, ab187978, Abcam), mouse-anti-Cyclin D1 (1:400, NBP2–32840, Novus Bio), rabbit-anti-AURKB (1:200, ab115793, Abcam), rabbit-anti-ki67 (1:100, ab16667, Abcam), rabbit-anti-p21 (1:200, ab188224, Abcam), rabbit-anti-p.H2AX(1:200, ab81299, Abcam), rat-anti-E-Cadherin (1:200, sc-59778, Santa Cruz Biotechnology, Inc.) guinea pig-anti-pan cytokeratin (1:50, BP5069, Origene). Primary antibody incubations were followed by 2 h incubation with the Polyclonal Goat Anti-Rabbit Immunoglobulins-HRP (1:200, P0448, Dako), Polyclonal Goat Anti-Mouse Immunoglobulins-HRP (1:100, P0447, Dako), goat anti-mouse [1:500, A32723, Invitrogen], goat anti-rabbit [1:500, A27039, Invitrogen], goat anti-guinea pig [1:500, ab175678, Abcam] or goat anti-rat [1:500, A-11006, Invitrogen]. For peroxidase reactions, colour was developed for 5 mins in DAB solution (Pierce™ 34002) and slides were counterstained in Mayer haematoxylin and DPX-mounted for light microscopy. Sections for immunofluorescence imaging were mounted using either: VECTASHIELD Antifade Mounting Medium with DAPI (Vector Laboratories) and imaged using a ZEISS Apotome microscope with ZEISS ZEN imaging software.

### Cell quantification

For bud area analysis, explant images were open in ImageJ, NIH^®^. The image scale was globally set according to the microscope magnifications used (10x for early explants and 5x for mature explants) and the bud area was outlined using the elliptical tool and measured using the “m” command. For the p.H2AX cell count, 7–9, random 20x fields (from three independent experiments) were captured and p.H2AX^+^ foci were manually counted using the cell counter command in ImageJ. For Cyclin d1 and ki67 percentage analysis: ki67^+^ or Cyclin D1^+^ cells were quantified in fifteen, 20x random fields (from three independent experiments) and percentage of positive nuclei for both markers within each field was calculated as: ki67^+^ or Cyclin D1^+^ cells/total cells X100. Values were analysed using GraphPad Prism and demonstrated as mean ± standard deviation (error bars).

### X-Gal Assay

The Explants at E13.5 were treated with DMSO or Baradertib as outlined above, after 24 h, the E14.5 glands were fixed in 4% paraformaldehyde for 20 min, then washed for 5 min in PBS supplemented with 1 mmol/L MgCl2, and stained for 5 to 6 h in PBS containing 1 mmol/L MgCl2, 1 mg/mL X-Gal, and 5 mmol/L of each potassium ferricyanide and potassium ferrocyanide. Accumulated spots of SA-β-Gal were counted in each explant and compared between the tested groups using GraphPad Prism.

### Measurement and inhibition of ROS

2′,7′-dichlorofluorescein diacetate (DCFDA) (ab113851, abcam) was used for the detection of the intracellular production of ROS in the E-SMGs. E16.5 explants were treated with DMSO or Barasertib for 24 h. The explants were harvested, washed once with PBS and exposed to 25 µM DCFDA for 1 h at 37 °C. Explants were washed twice with PBS and samples were analysed using a fluorescence microscope with filter set appropriate for fluorescein (FITC). Corrected Total Fluorescence (CTF) was analysed using ImageJ, NIH® and was calculated using the formula: CTF = Integrated Density − (Area of explant * Mean fluorescence of background). For inhibition of ROS, aminoguanidine (1 mM) was added either to Barasertib or DMSO prior to E-SMG incubations.

## Supplementary information

Supplementary Figure 1

Supplementary Figure 2

Supplementary Figure 3

Supplementary Figure 4
