# Supplementary figures and images for: Inhibition of Aurora Kinase B activity disrupts development and differentiation of salivary glands
Source: Cell Death Discov. 2021 Jan 18;7:16. doi: 10.1038/s41420-020-00393-w (PMC7814035; doi:10.1038/s41420-020-00393-w)

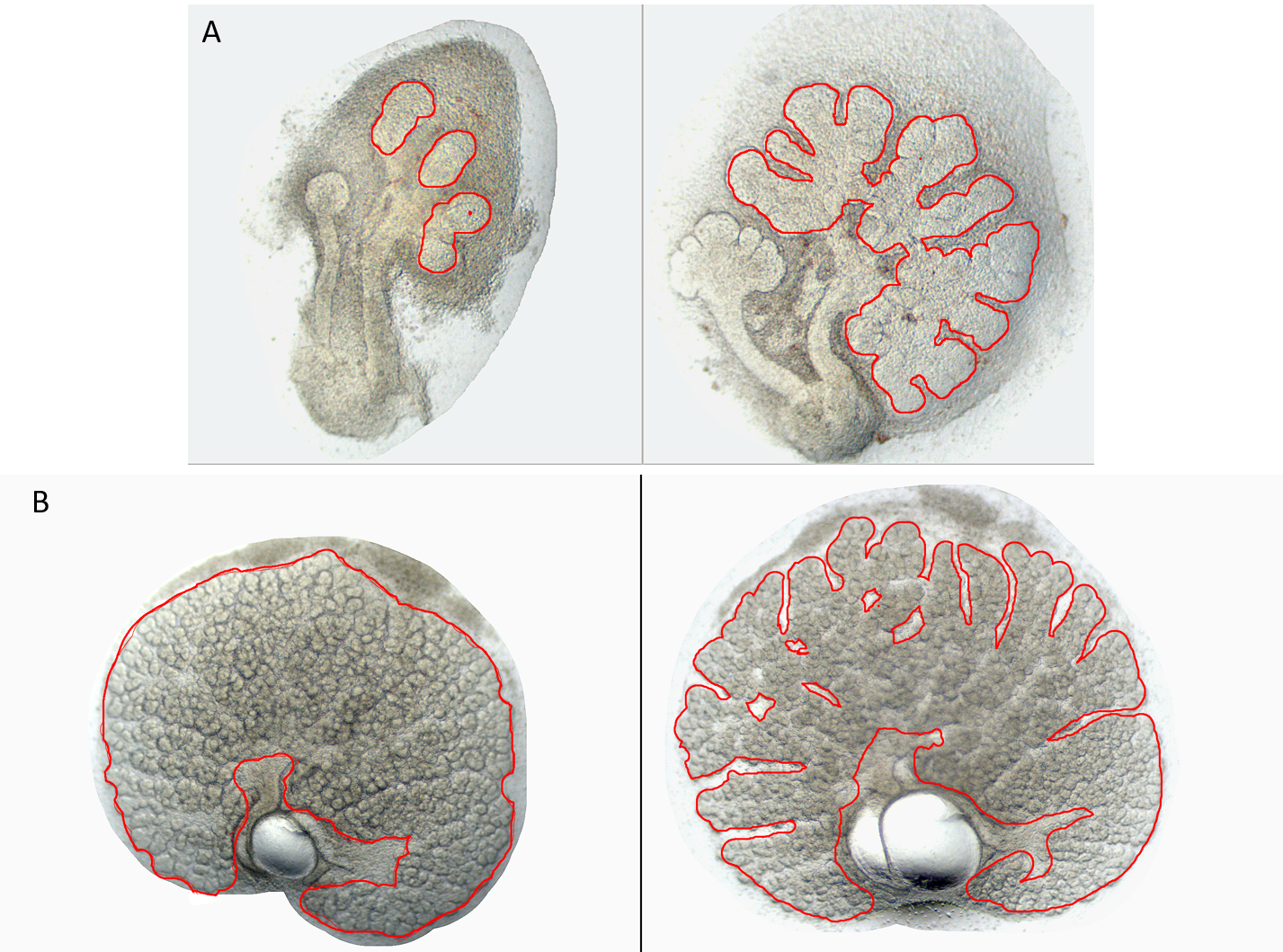

Supplement: Supplementary file 1 — Supplementary Figure 1 [file 41420_2020_393_MOESM1_ESM.png]

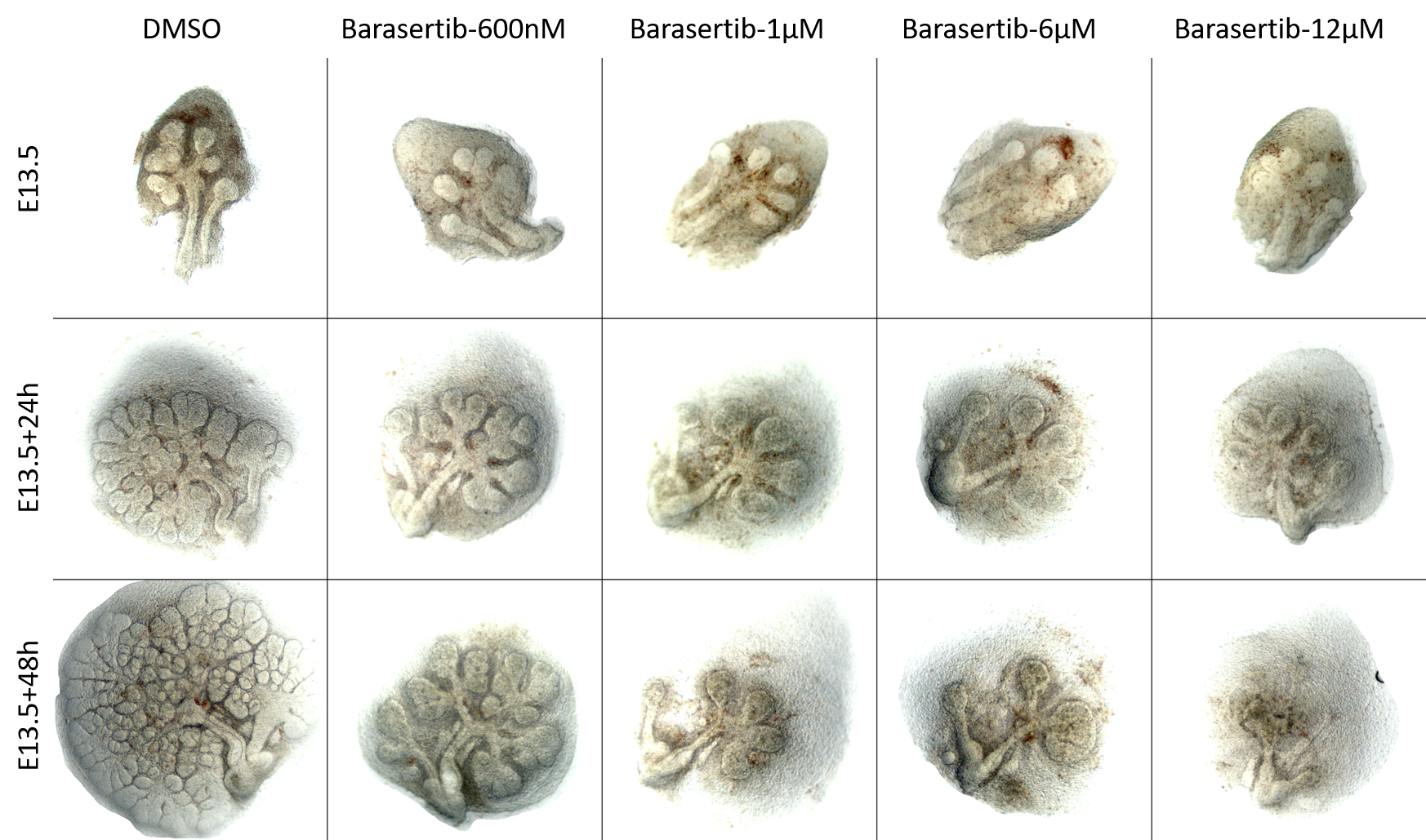

Supplement: Supplementary file 2 — Supplementary Figure 2 [file 41420_2020_393_MOESM2_ESM.png]

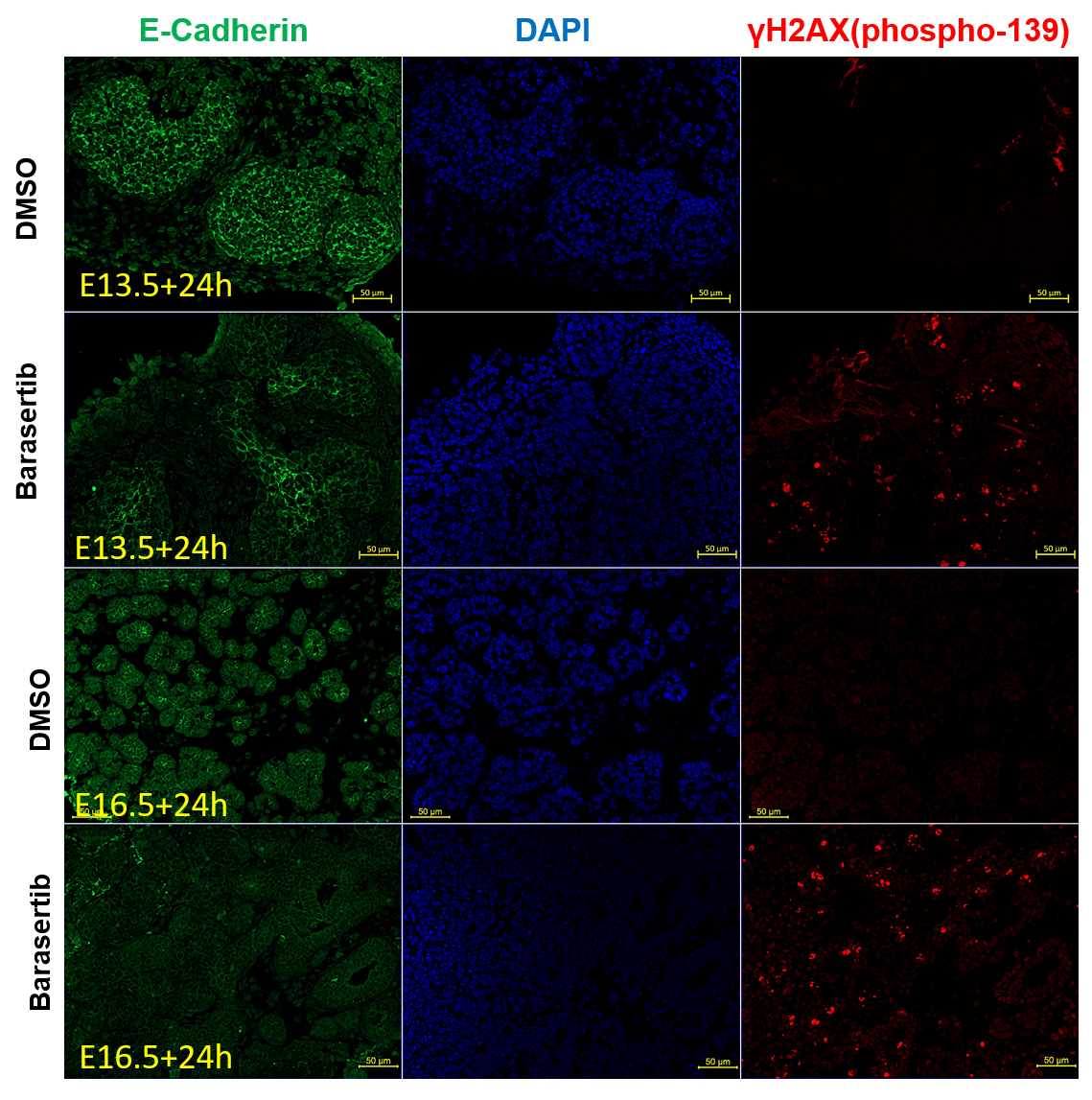

Supplement: Supplementary file 3 — Supplementary Figure 3 [file 41420_2020_393_MOESM3_ESM.png]

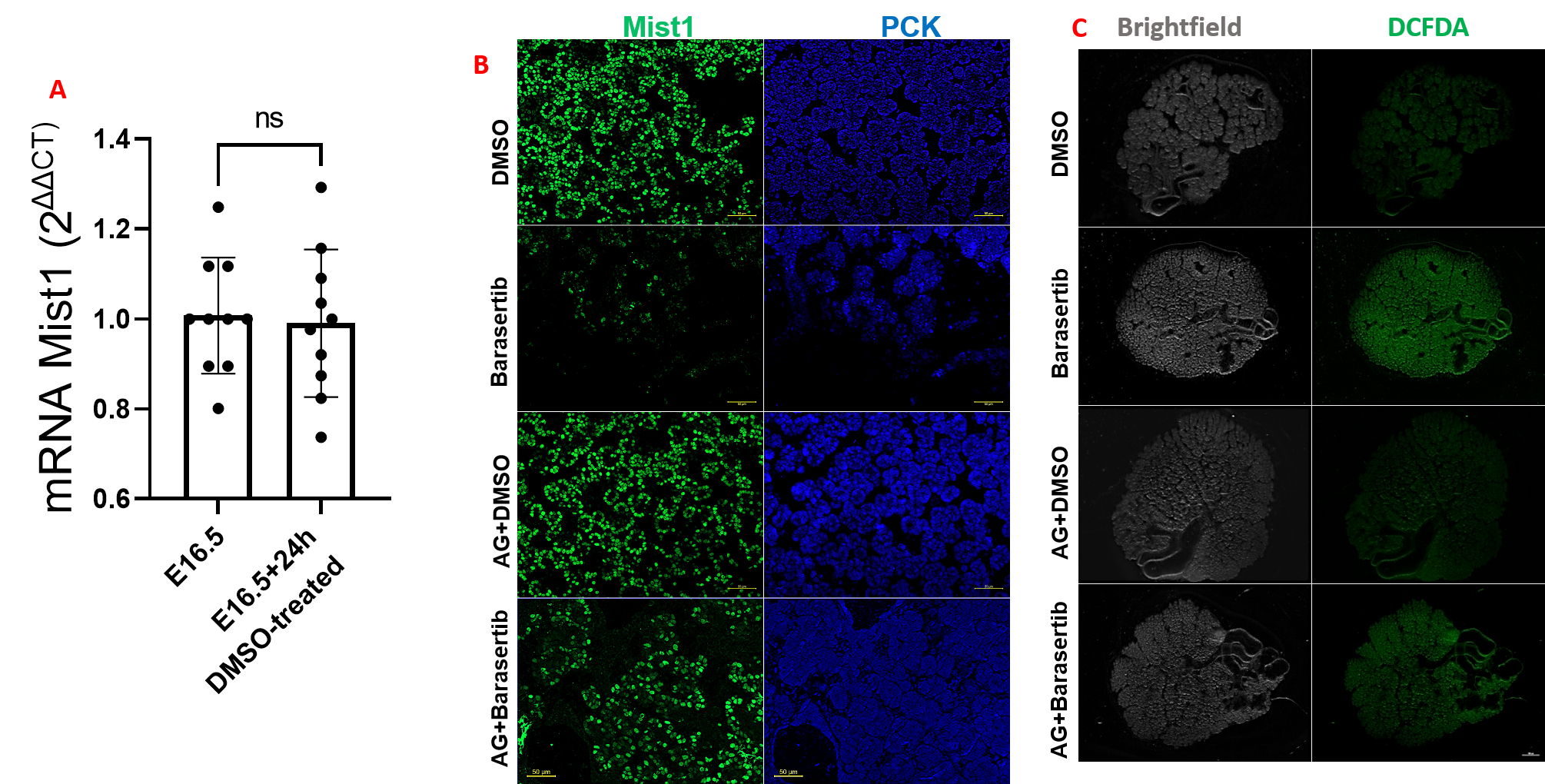

Supplement: Supplementary file 4 — Supplementary Figure 4 [file 41420_2020_393_MOESM4_ESM.png]
